# Coffee and Tea

**Published:** 1854-06

**Authors:** 


### COFFEE AND TEA.

In the January number, the moderate use of coffee and tea was advocated as healthful at regular meals, more so to invalids than cold water. In *Blackwood's Magazine*, for the same month, is an interesting paper, from which we learn that *black* and *green* tea are prepared from the *same* species of plant; the difference in color and in effects are produced by the modes of handling. For green tea, the leaves are roasted almost immediately after they are gathered. They are dried off quickly after the rolling process. For black tea they are allowed to be spread out in the air for some time after they are gathered. They are then further tossed about till they become soft and flaccid. They are now roasted for a few minutes and rolled, after which they are exposed to the air for some hours in a soft and moist state. Lastly, they are dried slowly over charcoal fires. The colored green teas are made by mixing Prussian blue and gypsum, and reducing them to a fine powder, which is applied to the teas during the process of wasting. The Chinese never drink these teas, and are much amused with the idea that the "outside barbarians" should prefer them to those of a natural green The best coffee grows on the driest soils. Yet the worst coffee, if kept ten or fourteen years, will acquire the flavor of the finest Mocha. The principal art in preparing coffee lies in roasting; for in this process it is that its peculiar aroma is produced. The heat should never be greater than is sufficient to impart to the berry a light brown color; for if carried beyond this point a disagreeable secondary smell mingles with the aroma. By the fashionable process of drinking coffee, that is, without the grounds, a good deal of nutritious matter is wasted, Many of the Oriental nations drink the grounds invariably. Not less than a hundred millions of the human race drink coffee, it is computed, as a daily beverage. In France, Germany, Sweden, Turkey, and a large portion of the United States, it is used by almost every body, just as tea is in England, Holland, Russia, and China. Experience, says the writer in *Blackwood,* teaches people that tea and coffee, used moderately, prevent the waste of the tissues, afford positive happiness, and increase the nervous activity, enabling men, as the writer in question, forcibly remarks, to throw more blood and spirit in the face of difficulties.

